# SENP2 regulates mitochondrial function and insulin secretion in pancreatic β cells

**DOI:** 10.1038/s12276-021-00723-7

**Published:** 2022-01-21

**Authors:** Jinyan Nan, Ji Seon Lee, Joon Ho Moon, Seung-Ah Lee, Young Joo Park, Dong-Sup Lee, Sung Soo Chung, Kyong Soo Park

**Affiliations:** 1 Department of Biomedical Sciences, Seoul National University College of Medicine, Seoul, Korea; 2 Department of Internal Medicine, Seoul National University College of Medicine, Seoul, Korea; 3 Genomic Medicine Institute, Seoul National University Medical Research Center, Seoul, Korea; 4 Department of Molecular Medicine and Biopharmaceutical Sciences, Graduate School of Convergence Science and Technology, College of Medicine, Seoul National University, Seoul, Korea; 5 Biomedical Research Institute, Seoul National University Hospital, Seoul, Korea

**Keywords:** Sumoylation, Diabetes, Mechanisms of disease, Phosphorylation

## Abstract

Increasing evidence has shown that small ubiquitin-like modifier (SUMO) modification plays an important role in metabolic regulation. We previously demonstrated that SUMO-specific protease 2 (SENP2) is involved in lipid metabolism in skeletal muscle and adipogenesis. In this study, we investigated the function of SENP2 in pancreatic β cells by generating a β cell-specific knockout (*Senp2*-βKO) mouse model. Glucose tolerance and insulin secretion were significantly impaired in the *Senp2*-βKO mice. In addition, glucose-stimulated insulin secretion (GSIS) was decreased in the islets of the *Senp2*-βKO mice without a significant change in insulin synthesis. Furthermore, islets of the *Senp2*-βKO mice exhibited enlarged mitochondria and lower oxygen consumption rates, accompanied by lower levels of S616 phosphorylated DRP1 (an active form of DRP1), a mitochondrial fission protein. Using a cell culture system of NIT-1, an islet β cell line, we found that increased SUMO2/3 conjugation to DRP1 due to SENP2 deficiency suppresses the phosphorylation of DRP1, which possibly induces mitochondrial dysfunction. In addition, SENP2 overexpression restored GSIS impairment induced by DRP1 knockdown and increased DRP1 phosphorylation. Furthermore, palmitate treatment decreased phosphorylated DRP1 and GSIS in β cells, which was rescued by SENP2 overexpression. These results suggest that SENP2 regulates mitochondrial function and insulin secretion at least in part by modulating the phosphorylation of DRP1 in pancreatic β cells.

## Glucose metabolism: regulation of insulin secretion

A protein called SENP2 plays a critical role in regulating secretion of insulin, the hormone that regulates blood sugar. Although SENP2 had been associated with type 2 diabetes, its role in regulating insulin secretion remained unclear. Kyong Soo Park and Sung Soo Chung at Seoul National University, South Korea, investigated how SENP2 regulates glucose metabolism using a mouse model that has SENP2-deficient pancreatic β cells. In the SENP2-deficient mice pancreatic β cells produced sufficient insulin but did not properly secrete it, and the cellular powerhouses, the mitochondria, were enlarged. Further investigation showed that SENP2 regulates a protein called DRP1 that triggers mitochondria to divide when they get too large. Boosting levels of SENP2 restored levels of functional DRP1 and boosted insulin secretion. These results improve our understanding of glucose metabolism, insulin-secreting cells, and diabetes.

## Introduction

Post-translational modification by small ubiquitin-like modifiers (SUMOs) occurs on specific regions of proteins to regulate various cellular processes by affecting their activity, localization, stability, or interactions with other proteins. This modification is reversed by SUMO/sentrin-specific proteases (SENPs)^1^. Increasing evidence suggests that SUMO modification also plays an important role in metabolic regulation^2^. In skeletal muscle, desumoylation of KLF5 by SENP1 modulates the PPARδ-dependent transcriptional program of fatty acid metabolism^3^. We also reported that fatty acids upregulate the expression of SENP2, which desumoylates PPARγ and PPARδ to increase the expression of fatty acid oxidation-related genes in skeletal muscle myotubes^4,5^. Consistent with this finding, muscle-specific SENP2 overexpression was shown to enhance fatty acid oxidation in skeletal muscle and protect mice from high-fat diet-induced insulin resistance^5^.

Several reports have demonstrated that SUMO modification is involved in the regulation of insulin synthesis and secretion in pancreatic β cells. Islet cell autoantigen 512 (ICA512) is an insulin granule protein, and after insulin exocytosis, the fragmented form of ICA512 translocates to the nucleus to enhance the transcription of insulin and granule proteins by binding to STAT5^6^. Sumoylation of ICA512 suppresses its binding to STAT5, resulting in decreased insulin synthesis^7^. SUMO1 overexpression in β cells inhibited Ca^++^-dependent exocytosis, which was rescued by SENP1 overexpression due to desumoylation of exocytosis-related proteins downstream of calcium influx^8,9^. In addition, islet-selective knockout of *Senp1* in mice disrupted insulin secretion, leading to impaired glucose tolerance^10^. These results demonstrate that SUMO modification and SENPs regulate insulin secretion in human β cells. Therefore, proteins targeted by SUMO modification should be identified to better understand the physiological regulation of insulin secretion.

Mitochondria play critical roles in insulin secretion in islet β cells^11,12^. Several mouse models with damaged β cell mitochondrial function show reduced insulin secretion and diabetic symptoms^13,14^. Mitochondria are dynamic organelles and undergo fission and selective fusion, allowing recovery or removal of damaged mitochondria in β cells^15^. In pancreatic β cells, inhibition of fission by knockdown of fission-related proteins, such as dynamin-related protein 1 (DRP1) and small fission protein 1 (FIS1), or by overexpression of a DRP1-dominant-negative mutant, decreased insulin secretion along with reduced mitochondrial autophagy^15–17^. In addition, *Drp1* knockout mice were found to be glucose intolerant on a normal chow diet, and in vivo glucose-stimulated insulin secretion (GSIS) and isolated islet GSIS were blunted^18^. In contrast, FIS1 overexpression generated fragmentation of mitochondria, which also resulted in a reduction in insulin secretion^19^. These reports demonstrate that an imbalance of fusion/fission induces defects in mitochondrial function, which results in impaired insulin secretion in islet β cells.

SENP2 expression was upregulated upon chronic glucose stimulation in INS1 cells and increased in the islets of an animal model of type 2 diabetes, as well as those from patients with type 2 diabetes mellitus^20^. However, the role of SENP2 in pancreatic β cells has yet to be investigated. Thus, in this study, we examined the involvement of SENP2 in glucose metabolism by generating pancreatic β cell-specific *Senp2* knockout (*Senp2*-βKO) mice. We found that these mice exhibited impaired insulin secretion accompanied by abnormal mitochondrial morphology and impaired mitochondrial function. Furthermore, our data reveal that SENP2 interacts with DRP1, and desumoylation of DRP1 promotes phosphorylation at S616 to potentiate insulin secretion under metabolic stress.

## Materials and methods

### Generation of pancreatic β cell-specific Senp2 knockout mice

Pancreatic β cell-specific Senp2 knockout mice (*Senp2*-βKO) were generated by mating *Senp2*-floxed mice (*Senp2*^*fl/fl*^) with RIP-Cre (rat insulin II promoter-Cre) mice. *Senp2*-floxed mice were generated by flanking exon 3 of the *Senp2* allele with LoxP (inGenious Targeting Laboratory, Stony Brook, NY, USA). All animal studies were performed in accordance with the Institutional Animal Care and Use Committee of Seoul National University Hospital.

### Animal experiments

The phenotypes of homozygous KO (*Senp2*^*fl/fl*^, RIP-Cre) male mice and their male littermates (*Senp2*^*fl/fl*^) were compared. Mice were fed a normal chow diet (CD) or a high-fat/high sucrose diet (HFD) (58 Kcal % fat with sucrose, Surwit Diet D12331) for 12–15 weeks from the age of 7–8 weeks. An intraperitoneal glucose tolerance test (IPGTT) was performed at the age of 20 weeks in the CD-fed mice and after 12 weeks of HFD feeding. Mice were fasted overnight for 16 h, and D-glucose (2 g/kg body weight for mice on a CD and 1 g/kg for mice on a HFD) was injected intraperitoneally. Blood glucose levels were measured from the tail vein using a glucometer (Accu-Chek, Roche, Basel, Switzerland). For in vivo glucose-stimulated insulin secretion (GSIS), insulin levels were measured from the serum collected at different time points during the IPGTT by an ultrasensitive insulin ELISA kit (ALPCO, Salem, OR, USA). Proinsulin and C peptide levels were measured by ELISA kits (ALPCO).

### Islet isolation and glucose-stimulated insulin secretion (GSIS)

Mouse islets were isolated in the conventional manner using collagenase as described previously^21^. Isolated islets were incubated for 3 h in RPMI media containing 10% FBS for further experiments. For ex vivo GSIS, islets were gently transferred in two wells of a 24-well noncoated plate. GSIS assays were performed in duplicate wells per mouse with normalization to the protein amount. Islets were rinsed with 2.8 mM glucose KRBH buffer (119 mM NaCl, 5 mM KCl, 2.5 mM CaCl_2_, 1.2 mM KH_2_PO_4_, 1.2 mM MgCl_2_, 10 mM HEPES, 25 mM NaHCO_3_, and 0.2% BSA) twice and then preadapted in low glucose KRBH buffer for 20 min. The islets were incubated with 2.8 mM glucose-containing KRBH buffer and incubated at 37 °C for 40 min to measure insulin secretion at low glucose. For high glucose stimulation, islets were incubated with 17.8 mM glucose-containing KRBH for 40 min. Insulin levels were measured from the supernatants. Islets were centrifuged and collected for protein quantitation. For measurement of insulin content in islets, ten islets per mouse were sonicated and incubated with acidic ethanol (0.2 M HCl in 95% ethanol) overnight at 4 °C. After neutralization with 0.2 M NaOH followed by centrifugation, insulin levels were measured by ELISA (ALPCO).

### Immunohistochemistry (IHC) and electron microscopy (EM)

Mouse pancreases were harvested after cardiac perfusion with PBS, fixed in 4% paraformaldehyde overnight, and then embedded in paraffin. Antibodies against SENP2 (Santa Cruz Biotechnology Inc., Dallas, TX, USA), insulin (Santa Cruz Biotechnology Inc.), and glucagon (Sigma-Aldrich, Saint Louis, MO, USA) were used for immunostaining. The ultrastructure of β cells was analyzed by transmission electron microscopy (TEM; JEM-100CX, JEOL). At least ten sections per mouse were selected and photographed in DG (40000X) by TEM. Insulin granules were classified according to their shapes: mature granules with electron-dense cores, immature granules with electron-translucent cores, and crystal granules with macroscopic crystals. Mitochondrial size was measured by the ECLIPSE Ci-L image program (Nikon, Tokyo, Japan).

### Measurement of oxygen consumption rate (OCR)

The OCR of islets or NIT-1 cells was analyzed by an XFe96 Seahorse bioanalyzer (Agilent Technologies, Santa Clara, CA, USA) according to the manufacturer’s protocol. The OCR of islets (15–20 islets/well) was analyzed in triplicate per mouse and normalized by protein content. Briefly, islets were incubated with assay medium (Seahorse XF base medium minimal DMEM, supplemented with 3 mM glucose and 0.2% BSA) for 1 h at 37 °C in a non-CO_2_ incubator. Islets were sequentially cultured in high glucose (20 mM), oligomycin (5 μM), FCCP (1 μM), and antimycin/rotenone (5 μM) for 20 min.

### Cell culture, siRNA, and plasmid transfection

NIT-1 cells were cultured in RPMI 1640 (Gibco, Thermo Fisher Scientific, Waltham, MA, USA) supplemented with 10% fetal bovine serum (FBS) and 1% penicillin-streptomycin at 37 °C and 5% CO_2_. NIT-1 cells were transfected with small interfering RNAs (siRNAs) against SENP2 (siSENP2, GE Healthcare Dharmacon, Lafayette, CO, USA) or DRP1 (siDRP1, Dharmacon) or nonspecific siRNA (negative control, Bioneer, Daejeon, Korea) using RNAiMAX (Invitrogen, Thermo Fisher Scientific, Waltham, MA, USA). The cells were used for GSIS or harvested for western blots 72 h after transfection. NIT-1 cells in 12-well plates were transfected with YFP-DRP1 (300 ng), UBC9 (500 ng), SUMO (500 ng), and SENP2 (200 ng) expression vectors using Lipofectamine and PLUS reagent (Invitrogen). The cells were harvested 48 h after transfection for western blot analysis. The YFP-DRP1 expression vector was a generous gift from Prof. W. Sun (Korea University, Korea). YFP-DRP1 mutants (K594R, K594/597 R) were generated using a site-directed mutagenesis kit (Agilent Technologies). The sequences of the primers used for mutagenesis are shown in Supplementary Table 1. For overexpression of SENP2, NIT-1 cells were treated with adenovirus (100 MOI) harboring the *Senp2* expression system (Ad-SENP2).

### RNA preparation and qPCR

Total RNA was extracted from isolated islets or NIT-1 cells using TRIzol (Invitrogen) according to the manufacturer’s instructions. Real-time qPCR was performed using SYBR-Master mix (TaKaRa, Shiga, Japan) and an ABI 7500 Real-time PCR system (Applied Biosystems, Foster City, CA, USA). The primers used for qPCR are listed in Supplementary Table 1.

### Immunoprecipitation

NIT-1 cells were transfected with siRNAs against SENP2 (siSENP2, Dharmacon) using RNAiMAX (Invitrogen). After 24 h, the cells in 6-well plates were transfected with YFP-DRP1 (200 ng), UBC9 (400 ng), and SUMO expression vectors (400 ng) using Lipofectamine and PLUS reagent (Invitrogen) for another 36 h. For palmitate treatment, NIT-1 cells were transfected with YFP-DRP1 (200 ng), UBC9 (400 ng), and SUMO expression vectors (400 ng) in 6-well plates. After 24 h, the cells were treated with 400 μM palmitate for another 24 h. Cell lysates were prepared with lysis buffer (20 mM Tris-HCl, pH 7.4, 1% NP-40, 10 mM Na_4_P_2_O_7_, 2 mM Na_3_VO_4_, 100 mM NaF, 5 mM EDTA, 7 μg/ml leupeptin, 7 μg/ml aprotinin and 1 mM PMSF). The cell lysates (400 μg) were used for immunoprecipitation with SUMO2/3 affinity beads (Cytoskeleton, Denver, CO, USA) for 16 h at 4 °C. The precipitates were washed three times with washing buffer (50 mM Tris pH 7.5, 150 mM NaCl, 1% IGEPAL, 20 mM NEM and protease inhibitor) and then washed two times with cold PBS. The beads were resuspended in 2x SDS-PAGE sampling buffer followed by heating for 5 min. After removal of agarose beads by centrifugation, the same amount of sample was subjected to SDS-PAGE and blotted with specific antibodies.

### Western blot analysis and antibodies

Proteins were extracted from isolated islets or NIT-1 cells using RIPA lysis buffer. Proteins (15–20 μg) were separated by SDS-PAGE and transferred onto nitrocellulose membranes (Whatman, Clifton, NJ, USA). After incubation with specific antibodies, the membrane bands were visualized by Amersham Imager 680 Blot and Gel Imagers (GE Healthcare Life Sciences, Marlborough, MA, USA). Antibodies against SENP2 (Santa Cruz Biotechnology Inc.), p616 DRP1 and DRP1 (Cell Signaling Technology, Danvers, MA, USA), GFP/YFP (Arigo Biolaboratories, Hsinchu City, Taiwan), GAPDH (Merck Millipore, Burlington, MA, USA), tubulin and Flag (Sigma-Aldrich) and OXPHOS Rodent WB antibody cocktail (Abcam, Cambridge, UK) were used for western blotting.

### Statistical analysis

Statistical analysis of the data was performed using Prism version 8. Student’s *t*-test (two-tailed) was used to measure the differences between two mean values. Data are expressed as the mean ± standard error, and data with a *P*-value < 0.05 were denoted as statistically significant.

## Results

### Generation of β cell-specific Senp2 knockout (Senp2-βKO) mice

**Fig. 1:**
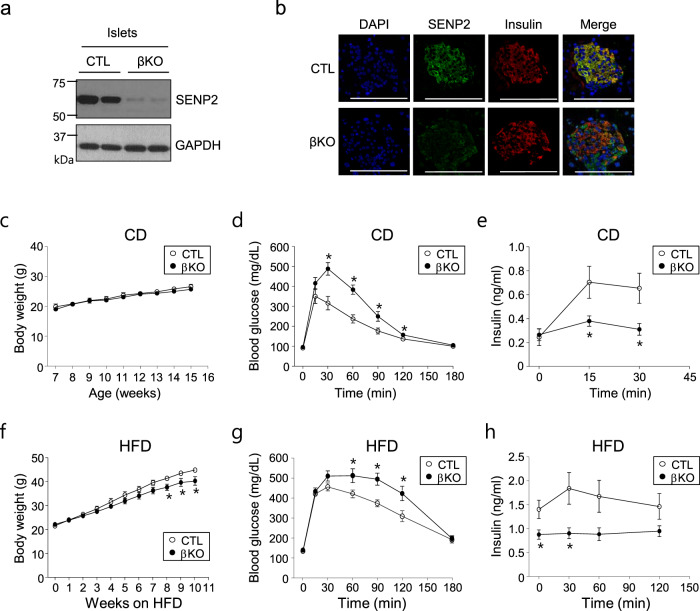
Glucose tolerance and insulin secretion in the *Senp2*-βKO mice. **a** Pancreatic islets were isolated from the control (CTL) or *Senp2*-βKO (βKO) mice, and then, SENP2 expression was detected by western blot analysis. **b** Pancreatic islets were subjected to immunofluorescence staining using antibodies against SENP2 and insulin; scale bar: 100 μm. **c**–**e** The control and *Senp2*-βKO mice were fed a control chow diet (CD). **c** Body weights. Serum glucose (**d**) and insulin levels (**e**) were measured at the indicated time points after glucose infusion (2 g/kg body weight). Data are presented as the mean ± SEM, *n* = 7 (CTL), 9 (βKO) (body weight), 5 (CTL), 6 (βKO) (GTT), and 6 (CTL), 7 (βKO) (insulin levels). *P < 0.05, *t*-test. **f**–**h** The control and *Senp2*-βKO mice were fed a high-fat diet (HFD). **f** Body weights. Serum glucose (**g**) and insulin levels (**h**) after glucose infusion (1 g/kg body weight). Data are presented as the mean ± SEM, *n* = 9 (CTL), 7 (βKO) (body weight, GTT), and 6 (serum insulin). *P < 0.05, *t*-test. All data are presented as the mean ± SEM.

*Senp2-*βKO mice were generated by breeding *Senp2*-floxed mice (*Senp2*^*fl/fl*^) with RIP-Cre mice. *Senp2*^*fl*/*fl*^ mice were used as controls (CTL). SENP2 was dramatically reduced in the islets of the *Senp2-*βKO mice but not in other tissues, including the hypothalamus (Fig. 1a and Supplementary Fig. 1a). SENP2 was specifically depleted in β cells of the Senp2-βKO mice, as confirmed by immunofluorescence staining; SENP2 was barely detected in β cells (insulin stained) but was easily observed in α cells (glucagon stained) in the islets of the *Senp2*-βKO mice (Fig. 1b and Supplementary Fig. 1b).

### Glucose tolerance and insulin secretion were impaired in the Senp2-βKO mice

To assess the effects of SENP2 deficiency on pancreatic β cells, we investigated the metabolic phenotypes of *Senp2-*βKO mice fed a standard chow diet (CD). Body weight and fasting glucose levels were comparable between the control and *Senp2-*βKO mice (Fig. 1c, d). However, glucose tolerance was significantly impaired in the *Senp2*-βKO mice after glucose infusion (2 g/kg) (Fig. 1d). The serum insulin concentration of the *Senp2*-βKO mice after glucose infusion was significantly lower than that of the control mice (Fig. 1e). When mice were fed a high-fat diet (HFD), weight gain was blunted in the *Senp2*-βKO mice without a significant reduction in food intake compared to that of the control mice (Fig. 1f and Supplementary Fig. 1c). The *Senp2*-βKO mice exhibited severe glucose intolerance compared to the control mice after 12 weeks of HFD feeding (Fig. 1g). Unlike that in the control mice, serum insulin was not increased at 30 min after glucose infusion in the *Senp2*-βKO mice (Fig. 1h). These results suggest that β cell-specific *Senp2* knockout results in impaired glucose tolerance due to dysregulation of insulin secretion.

### SENP2 deficiency affects insulin processing and glucose-stimulated insulin secretion

**Fig. 2:**
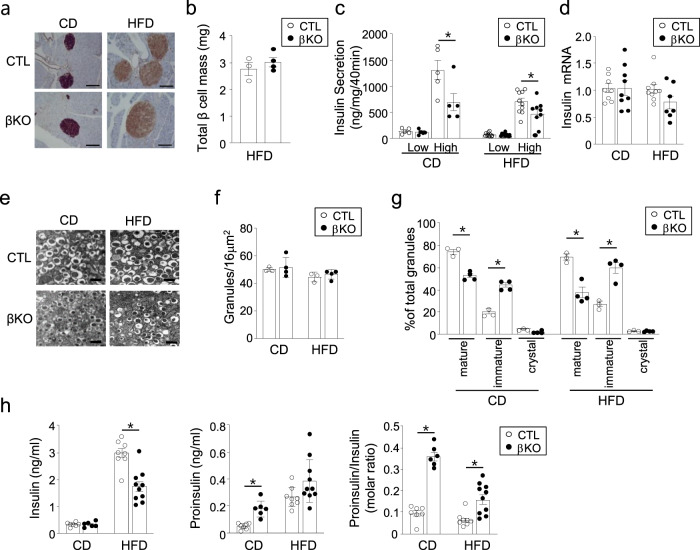
Pancreatic β cell-specific *Senp2* knockout affects insulin processing and maturation. **a** Immunohistochemical staining of the pancreas with insulin antibodies, scale bar: 100 μm. **b** Total β cell mass of the control and *Senp2*-βKO mice fed a 16-week HFD was measured with the following equation: β cell mass = pancreas weight × β cell area/pancreas area, which was determined by immunohistochemical staining with an insulin antibody. *n* = 3–4 mice. **c** GSIS of isolated islets from the control or *Senp2*-βKO mice. Islets were incubated under 2.8 mM (low) or 17.8 mM (high) glucose concentrations. *n* = 5–10 per group. *P < 0.05, *t*-test. **d** Relative insulin mRNA levels of the islets from the control and *Senp2*-βKO mice. The mRNA level of the control group was set to 1, and other values are expressed relative to this level. *n* = 7–9 per group. **e** Representative EM images (40000x) of isolated islets of the control and *Senp2*-βKO mice; scale bar: 500 nm. **f**, **g** Manual quantification and classification of insulin granules were performed on 5–6 areas/islet, >100 isolated islets/mouse. Total insulin granules per unit area (**f**) and distribution of insulin granule types (**g**), *n* = 3–4 mice per group, *P < 0.05, *t-*test. **h** Fasting serum insulin and proinsulin concentrations of the control and *Senp2*-βKO mice were measured after CD (20 weeks old) or 16 weeks of HFD (24 weeks old) feeding. Insulin (the left panel), proinsulin (the middle panel), and molar ratio of proinsulin to insulin (the right panel), *n* = 6–10 per group. *P < 0.05, *t-*test. All data are presented as the mean ± SEM.

To investigate the role of SENP2 in β cells, we evaluated insulin secretory capacity and processing in the islets of the *Senp2*-βKO and control mice. The islets in both groups showed compensatory mass expansion after HFD feeding, and the total β cell mass of the *Senp2*-βKO mice was comparable to that of the control mice (Fig. 2a, b). However, glucose-stimulated insulin secretion (GSIS) was significantly reduced in the islets of the *Senp2*-βKO mice compared to that of the control mice after both CD and HFD feeding (Fig. 2c). The insulin mRNA and protein levels in the isolated islets were similar between the control and *Senp2*-βKO mice (Fig. 2d and Supplementary Fig. 2), suggesting that SENP2 deficiency does not affect insulin synthesis. Based on the electron microscopic (EM) analysis of islet β cells, insulin granule numbers per unit area were similar between the control and *Senp2*-βKO mice (Fig. 2e, f). However, the proportion of immature granules was higher in the *Senp2*-βKO mice (Fig. 2g). Although the level of serum insulin in the *Senp2*-βKO mice was lower than that of the control mice on a HFD, the ratio of proinsulin to total insulin was higher in the serum of both the CD- and HFD-fed *Senp2*-βKO mice (Fig. 2h). Taken together, these data indicate that SENP2 plays important role in insulin processing and secretion in pancreatic β cells.

### SENP2 deficiency affects mitochondrial morphology and function in islet β cells

**Fig. 3:**
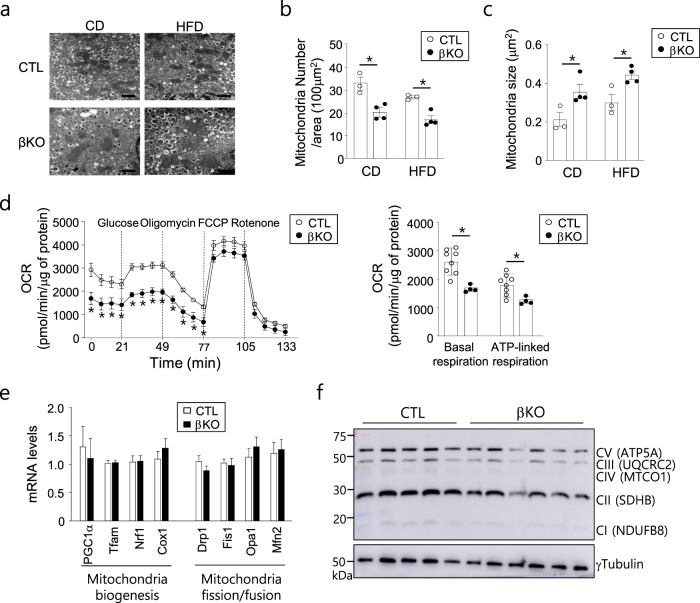
Effects of *Senp2* knockout on mitochondrial morphology and function of β cells. **a** EM images (x20000) of the isolated islets, scale bar: 1 μm, numbers of mitochondria per unit area (**b**), and average size of mitochondria (**c**). *n* = 3–4 mice per group, >70 mitochondria/mouse, *P < 0.05, *t-*test. **d** Oxygen consumption rates of the islets from the mice fed a CD. *n* = 8 (CTL), 4 (βKO), *P < 0.05, *t-*test. **e** Relative mRNA levels of the indicated genes in the islets of the control and *Senp2*-βKO mice on a CD. The mRNA level of the control group was set to 1, and the other values are expressed relative to this level. *n* = 8–15, *t-*test. **f** Western blot analysis was performed with islets isolated from the control and *Senp2*-βKO mice using antibodies against the indicated OXPHOS proteins. All data are presented as the mean ± SEM.

The mitochondrial morphology of β cells from the control or *Senp2*-βKO mice was analyzed by electron microscopy (EM). Enlarged or swollen mitochondria were observed more frequently in the β cells of the *Senp2*-βKO mice than in those of the control mice (Fig. 3a). The total number of mitochondria per unit area was lower, but the average size of the mitochondria was larger in the β cells of the *Senp2*-βKO mice than in those of the control mice (Fig. 3b, c).

To investigate the effect of SENP2 deficiency on mitochondrial function, we measured the oxygen consumption rate in islets isolated from 8-week-old control and *Senp2*-βKO mice. We found that both basal and ATP-linked respiration was significantly reduced in the islets of the *Senp2*-βKO mice (Fig. 3d). SENP2 deficiency did not alter the expression of genes related to mitochondrial biogenesis (*Tfam*, *Nrf1*, and *Cox1*) or mitochondrial fusion/fission (*Opa1*, *Mfn2*, *Drp1*, and *Fis1*) in the islets (Fig. 3e). However, several proteins that constitute the oxidative phosphorylation complex showed reduced expression in the islets of the *Senp2*-βKO mice (Fig. 3f and Supplementary Fig. 3). Taken together, these results demonstrated that SENP2 deficiency in islet β cells induces mitochondrial dysfunction with morphological changes in mitochondria.

### SENP2 deficiency affects the phosphorylation of DRP1 in pancreatic β cells

**Fig. 4:**
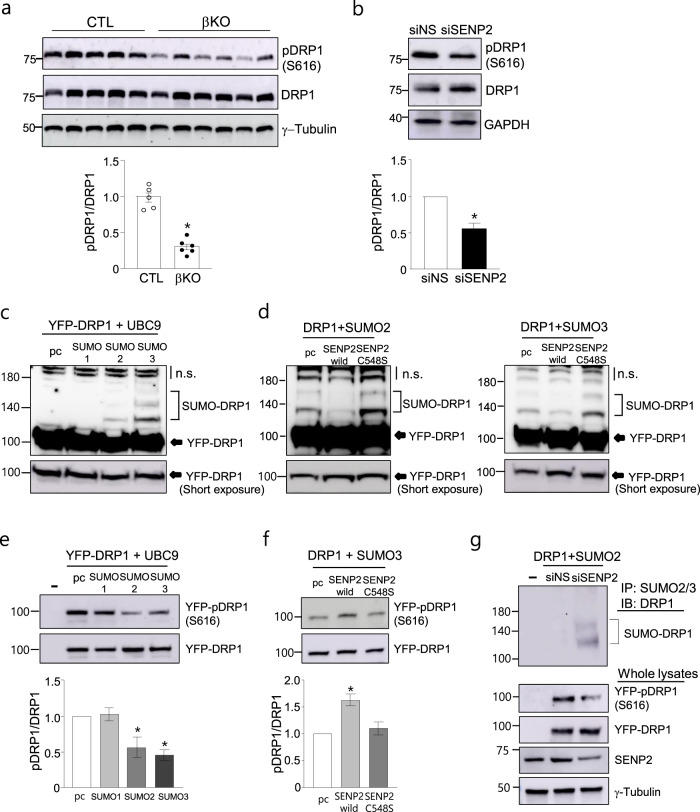
SUMO2/3 modification inhibits the phosphorylation of DRP1 in pancreatic β cells. **a** Western blot analysis was performed with islets isolated from the control or *Senp2*-βKO mice. Antibodies against pDRP1 (S616) and DRP1 (total DRP1) were used (the upper panel). The band densities were measured, and the ratio of pDRP1 to DRP1 of the control set was set to 1 (the lower panel). *n* = 5 (CTL) and 6 (βKO). *P < 0.05 vs. the control, *t-*test. **b** NIT-1 cells were transfected with siRNAs against SENP2 (siSENP2) or nonspecific control siRNAs (siNS). Cell lysates were used for western blot analysis (the upper panel). The ratio of pDRP1 to total DRP1 was measured (the lower panel). *n* = 4, *P < 0.05 vs. siNS, *t-*test. **c**, **d** NIT-1 cells were transfected with the expression vectors, and then, western blot analysis was performed using a DRP1 antibody 48 h after transfection. Cells were transfected with expression vectors for YFP-DRP1, UBC9, and SUMO1, −2 or −3 as indicated (**c**). Expression vectors for SENP2 (wild type) or SENP2 C548S (an inactive form) were transfected (**d**). pcDNAs (pc) were transfected as a control; nonspecific bands (n.s.). **e**, **f** After transfection of the indicated vectors, western blotting was performed with antibodies against pDRP1 (S616) and DRP1 (the upper panel), and then, the ratio of pDRP1 to total DRP1 was measured (the lower panel). The ratio in the absence of SUMO was set to 1, and the other values are expressed relative to this level (**e**). *n* = 4, *P < 0.05 vs. without SUMO, *t-test*. The ratio in the absence of SENP2 was set to 1, and the other values are expressed relative to this level (**f**). *n* = 4, *P < 0.05 vs. without SENP2, *t*-test. **g** NIT-1 cells were treated with siSENP2 and then transfected with YFP-DRP1 and SUMO2 expression vectors. Cell lysates were subjected to immunoprecipitation using a SUMO2/3 antibody 36 h after transfection. All data are presented as the mean ± SEM.

It has been reported that SENP2 desumoylates DRP1, a key component involved in mitochondrial fission^22^. In addition, knockdown of DRP1 in β cells or β cell-specific *Drp1* knockout decreased mitochondrial autophagy and impaired GSIS^15,16,18^. However, S616 phosphorylation of DRP1 has been demonstrated to activate DRP1 and increase the translocation of cytosolic DRP1 to the outer membrane of mitochondria (OMM), which facilitates mitochondrial fission in various cell types^23–25^. Therefore, we evaluated whether SENP2 is involved in the post-translational modification of DRP1, while DRP1 mRNA levels were not affected by *Senp2* knockout. When islets isolated from the control and *Senp2*-βKO mice were subjected to western blot analysis with an antibody specific to phosphorylated DRP1 at S616, the levels of phosphorylated DRP1 (pDRP1) in islets of the *Senp2*-βKO mice were lower than those of the control mice without any change in total DRP1 (Fig. 4a). We also examined the expression of other SENP isoforms in the control and *Senp2*-βKO islets, since DRP1 desumoylation by other isoforms, such as SENP3 and SENP5, has been reported^26^. The expression of other SENP isoforms, including SENP1, SENP3, and SENP5, was not affected by *Senp2* knockout (Supplementary Fig. 4a), excluding any effects on DRP1 phosphorylation by other SENPs. Similarly, phosphorylation of DRP1 at S616 was decreased by SENP2 knockdown using transfection of siRNAs against SENP2 (siSENP2) in NIT-1 cells (Fig. 4b and Supplementary Fig. 4b). These results suggest that SENP2 deficiency suppresses the phosphorylation of DRP1 at S616 in β cells, which possibly results in defects in mitochondrial function and β cell function.

### SUMO2/3 conjugation to DRP1 inhibits DRP1 phosphorylation at S616

Several reports have shown that DRP1 is a substrate of SUMO modification, and sumoylation affects the mitochondrial localization and function of DRP1 in various cell types;^27^ however, sumoylation of DRP1 in β cells has not yet been studied. Since we found that SENP2 deficiency affected the phosphorylation of DRP1, we hypothesized that sumoylation of DRP1 suppresses phosphorylation at S616 in DRP1. To investigate the relationship between sumoylation and phosphorylation of DRP1, we first transfected NIT-1 cells with vectors expressing YFP-DRP1, ubiquitin carrier protein 9 (UBC9, SUMO-conjugating enzyme), and one of the SUMO isoforms (SUMO1, −2 or −3). We found that DRP1 was efficiently sumoylated by SUMO2 and SUMO3 but not SUMO1 in NIT-1 cells (Fig. 4c). The lysine residue at the 597th amino acid of DRP1 (LKTSK^597^AEE) was identified as the major sumoylation site of DRP1 since little sumoylation by SUMO2 was observed with the DRP1 K597R mutant, in which the lysine residue was changed to arginine (Supplementary Fig. 4c). Wild-type SENP2, but not SENP2 C548S (an inactive form of SENP2), efficiently deconjugated SUMO2 and SUMO3 from DRP1 (Fig. 4d). Next, we measured phosphorylated DRP1 levels under different sumoylation conditions. Phosphorylation of DRP1 at S616 was decreased by overexpression of SUMO2 or SUMO3 (Fig. 4e). In addition, DRP1 phosphorylation was increased by coexpression of SENP2 but not by SENP2 C548S (Fig. 4f). Furthermore, the increase in sumoylation of DRP1 by SENP2 knockdown was confirmed using immunoprecipitation with a SUMO2/3 antibody and subsequent western blotting with a DRP1 antibody, and simultaneously, pDRP1 was decreased (Fig. 4g). These results consistently demonstrated that phosphorylation of DRP1 decreases when DRP1 sumoylation increases. Given that the K597 SUMO conjugation site is near the S616 phosphorylation site in DRP1, conjugation by SUMO2/3 may suppress the phosphorylation of DRP1 at S616 by interfering with the interaction of a kinase(s) with that site. Taken together, our results suggest that SENP2 plays a role in maintaining a pool of functional DRP1 by desumoylating DRP1.

### SENP2 overexpression restores DRP1 phosphorylation and insulin secretion in impaired β cells

**Fig. 5:**
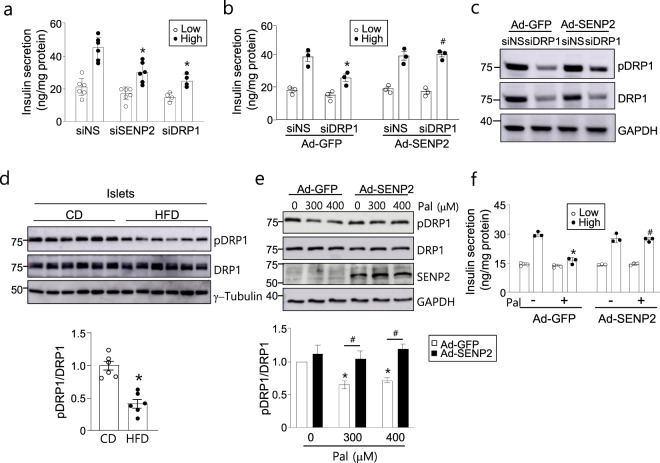
SENP2 overexpression restores DRP1 phosphorylation and insulin secretion. **a** GSIS was assessed 72 h after siRNA transfection in NIT-1 cells. *n* = 3–5. *P < 0.05 vs. siNS/high glucose, *t-*test. **b**, **c** NIT-1 cells were transfected with siDRP1, and after 4 h, the cells were infected with Ad-SENP2. GSIS was measured 2 days after virus infection. **b** Cells were incubated under low or high glucose concentrations. *n* = 3, *P < 0.05 vs. siNS/Ad-GFP (high), ^#^*P* < 0.05 vs. siDRP1/Ad-GFP (high), *t*-test. **c** Cell lysates were used for western blot analysis. **d** Islets were isolated from mice fed a CD or HFD for 20 weeks, and then, western blotting was performed. *n* = 6, *P < 0.05 vs. CD, *t*-test. **e** NIT-1 cells were infected with Ad-GFP or Ad-SENP2 for 24 h and then treated with palmitate for another 24 h. Western blotting was performed, and the ratio of pDRP1/DRP was measured. The pDRP1/DRP1 value of the Ad-GFP-infected cells in the absence of palmitate was set to 1. *n* = 3, *P < 0.05 vs. Pal (0 μM)/Ad-GFP, ^#^*P* < 0.05, *t*-test. **f** NIT-1 cells were infected with Ad-GFP or Ad-SENP2 for 24 h followed by palmitate (300 μM) treatment for 24 h, and then, GSIS was assessed. *n* = 3. *P < 0.05 vs. Pal (−)/Ad-GFP (high), ^#^*P* < 0.05 vs. Pal (+)/Ad-GFP (high), *t-*test. All data are presented as the mean ± SEM.

Next, we tested whether SENP2 knockdown affects insulin secretion in NIT-1 cells, as shown in islets of the *Senp2*-βKO mice. Knockdown of SENP2 and DRP1 significantly impaired GSIS in NIT-1 cells (Fig. 5a and Supplementary Fig. 5a). Interestingly, adenovirus-mediated overexpression of SENP2 recovered GSIS impairment by DRP1 knockdown (Fig. 5b and Supplementary Fig. 5b). Under the same conditions, phosphorylated DRP1 at S616 was found to be increased by SENP2 overexpression (Fig. 5c), suggesting that an increase in functional DRP1 (pDRP1) by SENP2 overexpression without any change in total DRP1 restores mitochondrial function for insulin secretion.

We also examined the role of SENP2 in DRP1 phosphorylation under lipotoxic conditions. Phosphorylated DRP1 levels in the islets of the HFD-fed mice were lower than those of the CD-fed mice without any change in total DRP1 (Fig. 5d). Similarly, palmitate treatment reduced DRP1 phosphorylation and impaired GSIS in NIT-1 cells (Fig. 5e, f). The increase in sumoylated DRP1 by palmitate treatment was also confirmed in NIT-1 cells transfected with YFP-DRP1 (Supplementary Fig. 5c). SENP2 overexpression facilitated the phosphorylation of DRP1 at S616 (Fig. 5e) and simultaneously restored palmitate-induced GSIS impairment (Fig. 5f). These results suggest that SENP2 overexpression ameliorates impaired β cell function during metabolic stress, and promoting DRP1 phosphorylation contributes to restoring β cell function, although SENP2 affects various proteins in addition to DRP1.

## Discussion

Here, we showed that the *Senp2*-βKO mice exhibited impaired insulin secretion and glucose intolerance accompanied by decreased mitochondrial function and abnormal mitochondrial morphology. In addition, we found that DRP1 is a substrate for SUMO2/3, and increased SUMO-conjugated DRP1 due to SENP2 deficiency suppresses the phosphorylation of DRP1 at S616 in pancreatic β cells. Overexpression of SENP2 restored DRP1 phosphorylation and β cell function impairment induced by palmitate treatment. Taken together, our data demonstrate that SENP2 regulates mitochondrial function and insulin secretion in pancreatic β cells, and modulation of DRP1 phosphorylation by SENP2 is one of the underlying mechanisms.

Mitochondrial function is crucial for insulin secretion in β cells^28^. Mitochondria provide ATP and other metabolic signals for glucose-coupled insulin secretion. Several studies have suggested that mitochondrial dysfunction is a major pathophysiological cause that impairs insulin secretion and induces insulin resistance in the development of type 2 diabetes^29,30^. Although the *Senp2*-KO β cells exhibited decreased oxygen consumption rates, reduced expression of some oxidative phosphorylation complex proteins, and enlarged mitochondria, no significant changes were observed in the expression of genes involved in mitochondrial biogenesis or dynamics (Fig. 3e). Thus, we focused on the mitochondrial fission protein DRP1, a SUMO target protein required for normal GSIS in pancreatic β cells^16,18,31^. We found that phosphorylation of DRP1 at S616 was significantly decreased in the *Senp2*-βKO islets, which was also confirmed in SENP2 knockdown-NIT-1 cells. However, SENP2 overexpression increased DRP1 phosphorylation in NIT-1 cells. Several reports have shown that DRP1 phosphorylation at S616 is important for regulating DRP1 translocation to mitochondria to initiate mitochondrial fission^25,32,33^. Thus, SENP2 could modulate DRP1 activity by affecting DRP1 phosphorylation at S616.

Several reports have demonstrated that sumoylation of DRP1 regulates its dynamic association with mitochondria in various cell types, including cardiomyocytes and neural cells. DRP1 can be modified by SUMO1 and SUMO2/3, and SUMO1 conjugation and SUMO2/3 conjugation differentially affect the mitochondrial association of DRP1^26,34^. SUMO1 conjugation stabilizes the DRP1 association with mitochondria, which enhances mitochondrial fragmentation and apoptosis^35,36^. In contrast, SUMO2/3 conjugation decreases the mitochondrial localization of DRP1^37,38^. These differences may be driven by the structural changes mediated by monosumoylation (SUMO1) versus branched polysumoylation (SUMO2/3). SENP3, SENP5, and SENP2 have been shown to deconjugate SUMOs from DRP1 in various cell types^26^. In the current study, we showed that DRP1 is efficiently sumoylated by SUMO2 and SUMO3 but not by SUMO1 in the pancreatic β cell line NIT-1. We also found that DRP1 modification by SUMO2/3 inhibits the phosphorylation of DRP1 at S616. Therefore, our results suggest that SENP2 deconjugates SUMO2/3 from DRP1 (at K597) and modulates DRP1 activity by increasing the phosphorylation of DRP1 at a nearby site (S616).

The relationship between phosphorylation and sumoylation has been extensively studied using phosphoproteome analysis in response to a decrease in SUMO levels, and the results showed that phosphorylation of some proteins is altered by their SUMO modification^39^. However, a phosphorylation-dependent SUMO modification motif (ψKxExxSP) has been identified in various proteins, and phosphorylation of the serine residue has been shown to promote sumoylation at the lysine residue within the motif^40^. These results clearly show interactions between sumoylation and phosphorylation in some proteins. Similarly, our study showed that sumoylation-modulated phosphorylation at S616 is involved in regulating DRP1 activity.

The insulin-resistant state of HFD models is accompanied by increased lipolysis and lipotoxicity. The HFD-fed *Senp2*-βKO mice barely showed an increase in insulin secretion after glucose stimulation. Moreover, the fasting insulin concentration was lower and the proportion of immature granules was higher in the *Senp2*-βKO mice. Intriguingly, we found that DRP1 phosphorylation at S616 decreased after HFD feeding and that palmitate treatment consistently decreased DRP1 phosphorylation in NIT-1 cells. However, SENP2 overexpression in NIT-1 cells rescued this phosphorylation and restored insulin secretion impaired by palmitate treatment. The role of DRP1 in insulin secretion in β cells has been intensively studied^15^. These studies have demonstrated that DRP1 is important for maintaining healthy mitochondria by eliminating damaged parts of mitochondria through a fission process. Given the importance of DRP1 in β cell function, maintaining the phosphorylated (active) DRP1 reservoir by increasing SENP2 activity or increasing the expression of SENP2 can be used as a therapeutic treatment to improve β cell function in lipotoxic conditions.

In addition to impaired GSIS, the *Senp2*-βKO mice showed higher serum proinsulin levels and more immature insulin granules than the control mice. These results suggest that insulin biosynthesis and secretory granule biogenesis are disrupted, which may be the result of mitochondrial dysfunction in the *Senp2*-KO β cells. Wikstrom et al. showed that DRP1 is found in the endoplasmic reticulum (ER), regulating ER morphology in stressed β cells and thereby modulating the β cell response to various metabolic stresses^41^. However, other explanations can be considered, including the possibility that SENP2 desumoylates other proteins that affect insulin processing and secretory pathways independent of mitochondrial function. It should be acknowledged that the potential effect of the human growth hormone cassette of RIP-Cre, which has been reported to impair glucose tolerance and insulin secretion, could not be excluded^42^.

In summary, SENP2 regulates mitochondrial function and insulin secretion in pancreatic β cells, and modulating sumoylation-mediated phosphorylation of DRP1 is an underlying mechanism. Future studies on the role of post-translational modification related to mitochondrial function in human β cells would provide additional insights into β cell physiology.

## Supplementary information

Supplemental Figures and Table
