# Supplementary material for: SENP2 regulates mitochondrial function and insulin secretion in pancreatic β cells
Source: Exp Mol Med. 2022 Jan 21;54(1):72–80. doi: 10.1038/s12276-021-00723-7 (PMC8814193; doi:10.1038/s12276-021-00723-7)
Supplement: Supplementary file 1 — Supplemental Figures and Table [file 12276_2021_723_MOESM1_ESM.pdf]

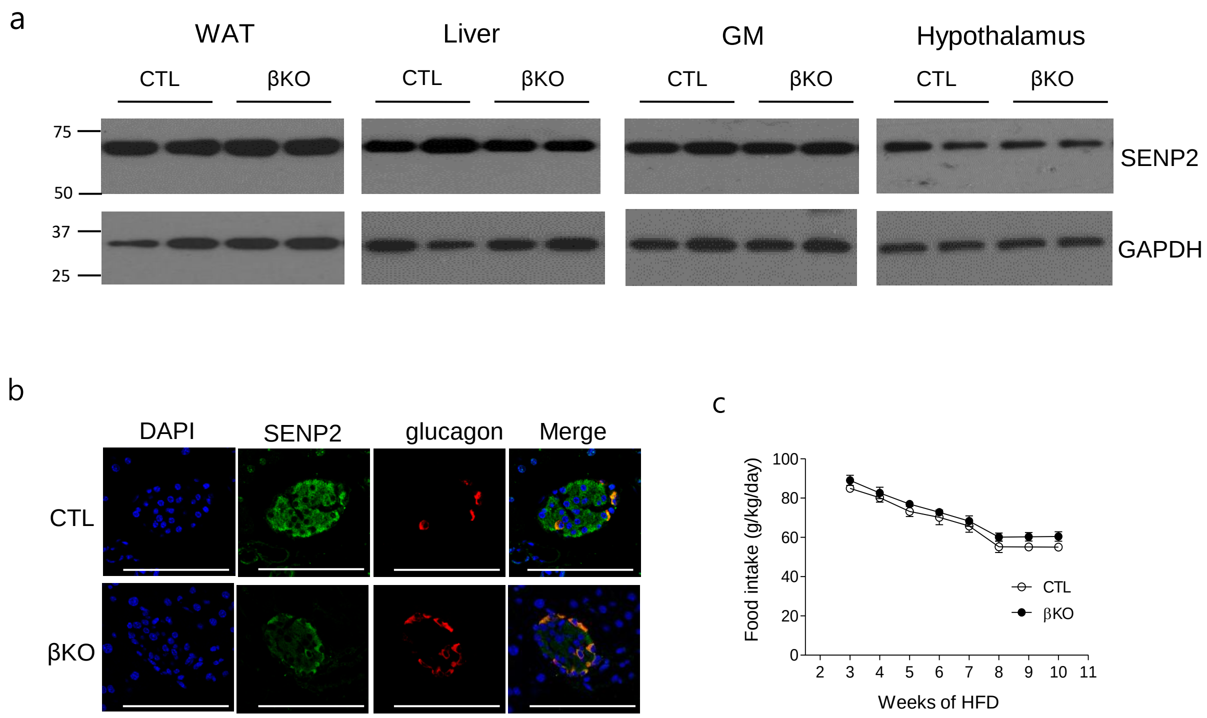

Supplementary Fig. 1 (a) Western blotting of SENP2 in various tissues of control and *Senp2*-βKO mice. (b) Immunofluorescence of SENP2 and glucagon in the islets of control and *Senp2*-βKO mice. (c) Food intake during HFD feeding.

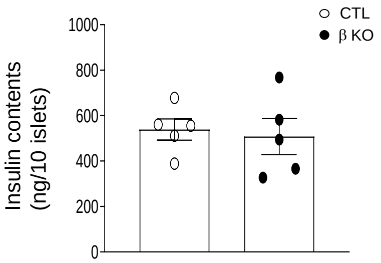

Supplementary Fig. 2 Insulin contents of islets isolated from control and *Senp2*-βKO fed a CD.

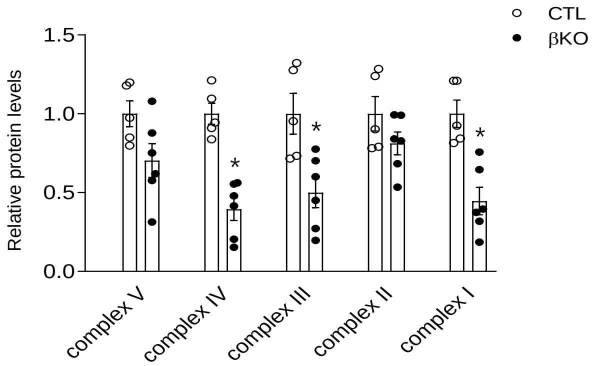

Supplementary Fig. 3 Relative amounts of the OXPHOS complex proteins in the western blot (Fig. 3f).

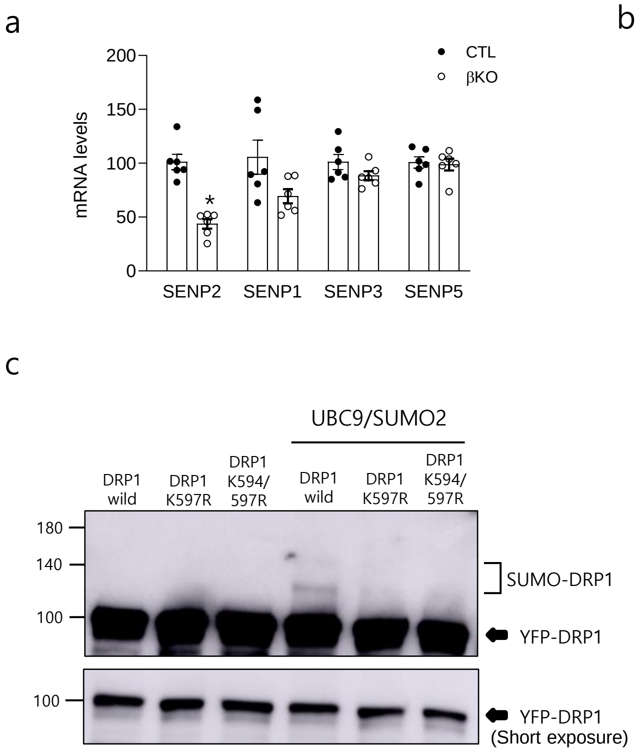

Supplementary Fig. 4 (a) The mRNA levels of SENP isoforms in the islets of *Senp2*-βKO were determined by qPCR. The value of control mice was set to 100 and the others were expressed as its relative. n =6, *P < 0.05 vs. CTL, *t*-test (b) Relative mRNA levels of SENP2 and DRP1 were determined 72 h after the siRNA transfection. The value of siNS was set to 1 and the other was expressed as its relative. n =3, *P < 0.05 vs. siNS, *t*-test. (c) NIT-1 cells were transfected with the expression vectors for YFP-DRP1 (wild type) or YFP-DRP1 mutants (K597R or K594/597R). An YFP antibody was used for western blot analysis.

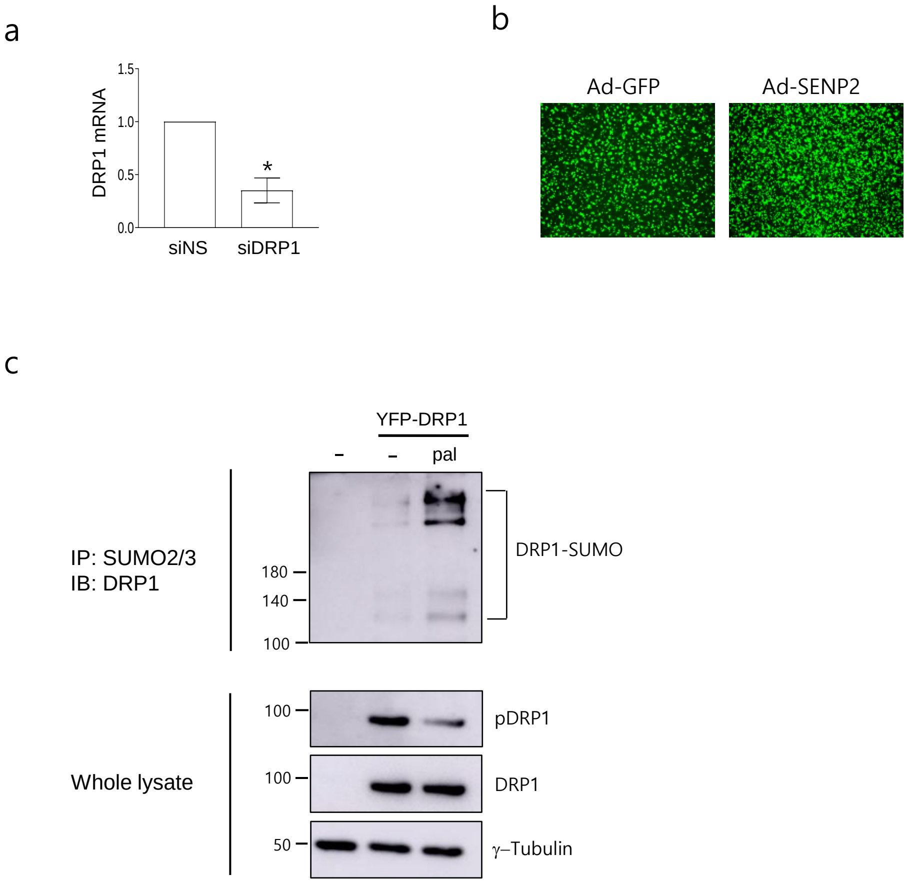

Supplementary Fig. 5 (a) Relative DRP1 mRNA levels after the transfection of siRNAs of DRP1 in NIT-1 cells. (b) GFP expression after the infection of Ad-GFP (control) or Ad-SENP2 into NIT-1 cells. (c) NIT-1 cells were transfected with expression vectors of YFP-DRP1, SUMO2/3 and UBC9, and then treated with palmitate (400 μM) for 24 h. Cell lysates were immunoprecipitated with a SUMO2/3 antibody and then immunoblotted with a DRP1 antibody.

Supplementary Table 1

	Forward	Reverse
Senp1	5′ CGT TCT TCC AGG CAG AGC TAT G 3′	5′ GCT GTA GTG CCA ATG CTT TCT GC 3′
Senp2	5′ CAG TCT CTA CAA TGC CAG 3′	5′ CCA GAA GGG GCC ACA TTC 3′
Senp3	5′ CTT ATG GCA GCC TCA TCC CTC T 3′	5′ TTG CCT GGC ATC CGC TGA TAA G 3′
Senp5	5′ TGG AAG TCT GGT CCC ACT CAG T 3′	5′ GGA AGT TAC ACT TTT GAT GTC TGG 3′
Pgc1α	5′ ACC TGA CAC AAC GCG GAC AG 3′	5′ TCT CAA GAG CAG CGA AAG CG 3′
Tfam	5′ GCT GAT GGG TAT GGA GAA G 3′	5′ GAG CCG AAT CAT CCT TTG C 3′
Nrf1	5′ TCG GGC ATT TAT CCC AGA GAT GCT 3′	5′ TAC GAG ATG AGC TAT ACT GTG TGT 3′
Cox1	5′ ACC ATC ATT TCT CCT TCT CC 3′	5′ GGT GGG TAG ACT GTT CAT CC 3′
Drp1	5′ GCG AAC CTT AGA ATC TGT GGA CC 3′	5′ CAG GCA CAA ATA AAG CAG GAC GG 3′
Fis1	5′ GCT GGT TCT GTG TCC AAG AGC A 3′	5′ GAC ATA GTC CCG CTG TTC CTC T 3′
Opa1	5′ TCT CAG CCT TGC TGT GTC AGA C 3′	5′ TTC CGT CTC TAG GTT AAA GCG CG 3′
Mfn2	5′ GTG GAA TAC GCC AGT GAG AAG C 3′	5′ CAA CTT GCT GGC ACA GAT GAG C 3′
Gapdh	5′ AGG TCG GTG TGA ACG GAT TTG 3′	5′ TGT AGA CCA TGT AGT TGA GGT CA 3′
β-actin	5′ GAA GCT GTG CTA TGT TGC TCT A 3′	5′ GGA GGA AGA GGA TGC GGC A 3′
YFP-DRP1 K597R	5′ CTG GAG AGG AAT GCT GAA AAC TTC ACG TGC TGA AGA G 3′	5′ CTC TTC AGC ACG TGA AGT TTT CAG CAT TCC TCT CCA G 3′
YFP-DRP1 K594/597R	5′ GGA GAG GAA TGC TGC GTA CTT CAC GTG CTG AAG AG 3′	5′ CTC TTC AGC ACG TGA AGT ACG CAG CAT TCC TCT CC 3′
